# GRK2 Mediated Abnormal Transduction of PGE2-EP4-cAMP-CREB Signaling Induces the Imbalance of Macrophages Polarization in Collagen-Induced Arthritis Mice

**DOI:** 10.3390/cells8121596

**Published:** 2019-12-08

**Authors:** Xuezhi Yang, Susu Li, Yingjie Zhao, Siyu Li, Tianjiao Zhao, Yu Tai, Bingjie Zhang, Xinwei Wang, Chun Wang, Jingyu Chen, Qingtong Wang, Lingling Zhang, Dexiang Xu, Yan Chang, Wei Wei

**Affiliations:** 1 Institute of Clinical Pharmacology, Anhui Medical University, Key Laboratory of Anti-Inflammatory and Immune Medicine (Anhui Medical University), Ministry of Education, Anhui Collaborative Innovation Center of Anti-inflammatory and Immune Medicine, Hefei 230032, China; leoyxz@126.com (X.Y.); 18895314362@163.com (S.L.); zyjfy2011@163.com (Y.Z.); lisiyu1011@163.com (S.L.); ztjexp@163.com (T.Z.); taiyu0331@163.com (Y.T.); 18297989420@163.com (B.Z.); wangxw2019@126.com (X.W.); wangchun@ahmu.edu.cn (C.W.); cjyanyi@126.com (J.C.); hfwqt727@163.com (Q.W.); ll-zhang@hotmail.com (L.Z.); 2 Public Health and Preventive Medicine Postdoctoral Research Station of Anhui Medical University, Hefei 230032, China

**Keywords:** PGE2, GRK2, macrophage polarization, cAMP, EP4

## Abstract

Rheumatoid arthritis (RA) is characterized by the massive infiltration of various chronic inflammatory cells in synovia. In synovial fluid of patients with RA, M1 macrophages are dominant among all subtypes of macrophages, the mechanisms of macrophages polarization imbalance in RA has not been fully illuminated. The prostaglandin E2 (PGE2) augments M2 polarization in part via the cyclic adenosine monophosphate (cAMP)-cyclic AMP responsive element binding (CREB) signaling. However, previous study found constant stimulus of PGE2 on fibroblast-like synovial cells of adjuvant arthritis rats induced the decrease of cAMP, which is primarily caused by G protein-coupled receptor kinase 2 (GRK2)-induced EP4 over- desensitization. Whether GRK2 mediated-EP4 over-desensitization reduces the level of cAMP and inhibits M2 polarization in RA is unclear. Here we observed M1 macrophages were dominant in peritoneal macrophages (PMs), bone-marrow-derived macrophages (BMMs) and synovial macrophages of collagen-induced arthritis (CIA) mice. PGE2 stimulated M2 polarization via the EP4-cAMP-CREB in normal mice, while failed to promote M2 polarization in the PMs of CIA mice. Further, we found the EP4 over-desensitization stimulated by PGE2 induced abnormal PGE2-cAMP-CREB signaling as well as the imbalance of macrophage polarization. Targeted disruption of GRK2 in Raw264.7 (RAW) through GRK2 siRNA or CRISPR/Cas9 downregulated the M1 macrophage markers, upregulated the M2 macrophage markers and the EP4 membrane localization. The reduced M1/M2 ratio and increased p-CREB expression were observed in BMMs and PMs of GRK2^+/−^ mice. This study highlighted a novel role of GRK2 in regulating macrophages function in RA and provided new idea for precision treatment of RA.

## 1. Introduction

Rheumatoid arthritis (RA) is a systemic autoimmune disease, with major pathological features including synovitis and cartilage destruction [1,2,3,4]. The degree of synovial macrophage infiltration correlates with the degree of joint erosion, and depletion of these macrophages from inflamed tissue has a profound therapeutic benefit. According to the phenotype and function, macrophages are mainly divided into M1 macrophages (as proinflammatory) and M2 macrophages (as anti-inflammatory). Under normal physiological conditions, the phenotypes of these macrophages keep dynamic balance and maintain homeostasis. In RA patients and animal models of arthritis, multiple factors bring the predominant position to M1 macrophages in peritoneal blood, peritoneal fluid and synovium [5]. M1 macrophages in turn aggravate inflammation by producing inflammatory factors which activate fibroblast-like synovial cell (FLS) proliferation and Th17 polarization [6]. However, the mechanisms of polarization imbalance of macrophages in RA have not been elucidated.

Prostaglandin E2 (PGE2) is a potent lipid mediator with significantly increased level in serum and synovium of RA. PGE2 mediates inflammation through its four PGE2 receptors (EP)(EP1-4). Reportedly, PGE2 augments M2 polarization in part via the cyclic adenosine monophosphate (cAMP)-cyclic AMP responsive element binding (CREB) signaling [7]. However, the role of PGE2 on macrophages polarization in RA and which EP receptor plays a role in macrophages polarization are still not systematically investigated.

EP receptors are G protein-coupled receptors (GPCRs), activated GPCRs can be phosphorylated by G protein-coupled receptor kinases (GRKs). GRK2 is a ubiquitous, essential protein kinase that is emerging as an integrative node in many signaling networks. Accumulating data indicate that GRK2 is overexpressed in RA patients and animal models [8,9,10], targeting GRK2 plays a critical role in ameliorating inflammatory pain [11] and duration of mechanical hyperalgesia [12]. Our previous study found that the GRK2 inhibitor paroxetine can inhibit T cell infiltration to the synovial tissue of rats with collagen-induced arthritis (CIA) [13]. However, the regulatory effect of GRK2 on macrophages in RA has not been reported.

GRKs are identified as key players in the desensitization and internalization of multiple GPCRs. The role of GRKs-induced GPCRs excessive desensitization in heart failure was extensively described [14]. Excessive desensitization of β-adrenergic receptors mediated by GRK2 resulted in a loss of cardiomyocyte contractile reserve. In RA, our previous study confirmed that in the FLS of adjuvant induced arthritis (AA) rats, continuous stimulation of PGE2 promoted GRK2 transferring to membrane [13,15,16], which induced the over-desensitization of EP4 and downregulated the level of cAMP [15]. Based on these above studies, we speculated that in the macrophages of RA, continuous stimulation of PGE2 on EP4 may result in the translocation of GRK2 to membrane, leading to the over-desensitization of EP4, reduction of cAMP.

Here, we explored the ratio of M1/M2 markers in peritoneal macrophages (PMs), bone-marrow-derived macrophages (BMMs) and synovial macrophages (SMs), PGE2-stimulated macrophage polarization, EP1-EP4 membrane localization and PGE2-cAMP-CREB signaling in CIA mice. We found PGE2 failed to promote M2 polarization in CIA mice, but increased M2 mark expression in PMs of normal mice. The over-desensitization of EP4 stimulated by PGE2 results in abnormal PGE2-cAMP-CREB signaling as well as imbalance of macrophage polarization in CIA mice. Targeted disruption of GRK2 in Raw264.7 cells (RAW) through GRK2 siRNA or CRISPR/Cas9 downregulated M1 marker expression and upregulated M2 marker expression and EP4 membrane localization. The downregulated M1/M2 ratio were also shown in macrophages of GRK2^+/−^ mice, as well as upregulated p-CREB expression. Our results suggested a novel role for GRK2 in regulating macrophage polarization, targeting GRK2 showed a kind of potential for treating patients with RA.

## 2. Materials and Methods

### 2.1. Animals

DBA/1 mice, with the weight of 18 g ± 4 g, were purchased from Shanghai SLAC Laboratory Animal Co. Ltd. (Shanghai, China). C57/6J mice, with the weight of 22 ± 4 g, were purchased from Animal Centre of Anhui Medical University (Hefei, Anhui Province, China). GRK2^+/−^ mice, with the weight of 22 ± 4 g, were design by our group and constructed by Model Animal Research Center of Nanjing University (Nanjing, Jiangsu Province, China).

GRK2 gene of mice was located on chromosome 19 with gene ID 11035, including 21 exons. According to the design principles of sgRNA, exons 3, 4 and 5 were selected as the design targets. A pair of sgRNAs were screened by using the CRISPR Design software of MIT (http://crispr.mit.edu). The sgRNA sequences of GRK2: GRK2-S1 (GGCAGAAGTCCCGGAAAAGC), GRK2-S2 (TCTGGAAGAGGCCAAGCCCT). The adjacent motifs of the antecedent sequence were identified as AGG and TGG. The designed sgRNA oligonucleotide single strand was anneal by PCR machine to form a double strand, then the double-stranded sgRNA was connected with the PGK1.1 linear vector at 16 °C overnight under the action of T4 DNA ligase, and the ligation product was transfected into DH5a competent cells. Positive clones were selected through PCR and gene sequencing to complete the construction of sgRNA vector. In vitro transcription of sgRNA mixed with Cas9 mRNA was injected into the fertilized eggs of C57BL/6 mice, and the fertilized eggs were transplanted into the female C57BL/6 mice to produce F0 generation mice. F1 generation mice: Female F0 generation GRK2^+/−^ mice and C57BL/6 male mice were caged at 1:1, and the gestation period of pregnant mice was generally about 21 days. F1 generation GRK2^+/−^ mice with the 19bp deletion were mated with C57BL/6 mice and obtained F2 generation GRK2^+/−^ mice with good stable heritability. Mice tail DNA was extracted for PCR identification (unpublished). All experiments were approved by the Ethics Review Committee for Animal Experimentation of Institute of Clinical Pharmacology, Anhui Medical University.

### 2.2. Reagents

Chicken type II collagen (CII): Chondrex, Inc. (Waltham, MA, USA). Dulbecco’s modified Eagle’s medium (DMEM) and fetal bovine serum (FBS): Gibco Co. (Grand Island, NY, USA). Lipopolysaccharides (LPS): Sigma Chemical Co. (St. Louis, MO, USA). PGE2 (purity ≥ 98%) and CAY10598 (EP4 agonist): Cayman Chemical Company (Ann Arbor, MI, USA). GSK180736A (GRK2 inhibitor), dbcAMP (cAMP analogue) and PF-04217329 (EP2 agonist): MedChemExpress (Monmouth Junction, NJ, USA). PGE2 high sensitivity ELISA kit: Enzo Biochem (Farmingdale, NY, USA). cAMP Activity Assay Kit: BioVision (Milpitas, CA, USA). ATP1A1 rabbit polyclonal antibody and iNOS rabbit polyclonal antibody: Proteintech Group, Inc. (Chicago, IL, USA). Rabbit anti-mouse GRK2, rabbit anti-mouse EP2, rabbit anti-mouse EP3, rabbit anti-mouse EP4 and protein A/G plus agarose: Santa Cruz Biotechnology, Inc. (Santa Cruz, CA, USA). Arg1 rabbit mAb, p-CREB rabbit mAb and CREB rabbit mAb: Cell Signaling Technology, Inc. (Danvers, MA, USA). APC-F4/80: Miltenyi. PE-CD86 and APC-CD206: eBioscience, Inc. (San Diego, CA, USA). Mouse GRK2 siRNA: Shanghai Genepharma Co., Ltd. (Shanghai, China). Mouse GRK2 CRISPR/Cas9: Shanghai Genechem Co., Ltd. (Shanghai, China). Recombinant mouse TNF-α, recombinant mouse IFN-γ and recombinant mouse IL-4: PeproTech Co. (Rocky Hill, NJ, USA). Nuclear and cytoplasmic protein extraction kits: Beyotime (Shanghai, China).

### 2.3. The Establishment of CIA Model

C II was dissolved with 0.1 M of acetic acid and then emulsified with equivalent amount of complete Freud’s adjuvant (CFA). CFA is purchased from sigma. The final concentration of the reagent was 2 mg/mL. After overnight storage at 4 °C, 0.1 mL reagent was injected into the bottom of the tail of DBA/1 mice, and the same dose was injected into the back again after 21 days [17]. Mice were divided into normal group and CIA model group (*n* = 8 per group). The normal and CIA mice were given an equal volume of vehicle.

### 2.4. Cells Isolation and Cell Culture

PMs were isolated from peritoneal fluid of mice. PMs were plated into sterile Petri dishes and incubated in DMEM supplemented with 10% FBS. PMs were incubated at 37 °C with 5% CO_2_ and harvested after 2 h [16]. BMMs were isolated from the femurs of mice. BMMs were plated into sterile Petri dishes and incubated in DMEM supplemented with 10% FBS and 10% macrophage colony-stimulating factor (M-CSF)-conditioned media. BMMs were incubated at 37 °C with 5% CO_2_ and harvested after 7 d [18]. SMs were isolated from the synovium of mice. SMs were plated into DMEM (+5% FBS) containing 1 mg/mL type Ⅳ collagenase and incubate for 1.5 h with shaking. After incubation, cells were collected by centrifugation (2500 rpm, 5 min) [19]. RAW macrophages and constructed GRK2 KO macrophages were cultured as previously described [20]. RAW macrophages were purchased from ATCC (Manassas, VA, USA) and incubated at 37 °C with 5% CO_2_. We stimulated RAW into M1 macrophages through TNF-α (50 ng/mL, 24 h) and IFN-γ (50 ng/mL, 24 h). We stimulated RAW into M2 macrophages through IL-4 (20 ng/mL, 24 h).

### 2.5. Evaluation of Arthritis

An evaluation of the severity of the CIA was performed by two independent observers with no knowledge of the treatment protocol. Beginning on day 21 after immunization, the mice were evaluated every 3 days using arthritis index (AI) assessment. After the onset of inflammation, the AI of the CIA mice in each group was evaluated once every 3 days as follows: 0, no signs of arthritis; 1, swelling and/or redness of the paw or one digit; 2, two joints involved; 3, more than two joints involved; and 4, severe arthritis of the entire paw and all digits. All feet were measured, the maximum score value of each mouse was 16 [21].

### 2.6. Protein Sample Preparation

The total protein preparation: PMs, BMMs and RAW were lysed and centrifuged at 14,000× *g* for 15 min at 4 °C. Collecting the supernatant and added the protein loading buffer (5×), then the sample was boiled for 8 min. These samples were used to detect the expression of EP1-EP4, iNOS, Arg1, p-CREB, CREB and β-actin [16].

Membrane protein expression: PMs, BMMs and RAW were lysed and centrifuged at 14,000× *g* for 15 min at 4 °C. Collecting the supernatant and centrifuged at 100,000× *g* for 1 h at 4 °C. Removing the supernatant, the precipitated membrane protein was resuspended by 50 μL cell lysis buffer and 10 μL protein loading buffer (5×), then the sample was boiled for 5 min. These samples were used to detect the membrane expression of EP4, GRK2 and ATPA1 [15,16].

### 2.7. Western Blot Analyses

The denatured protein was separated by 10% SDS-PGE and transferred electrophoretic ally to a polyvinylidene fluoride membrane. The dilution of primary antibody of EP1-EP4, iNOS, Arg1, p-CREB, CREB, β-actin and ATPA1 is 1:1000. The dilution of second primary antibody of goat anti-mouse is 1:30,000, of goat anti-rabbit is 1:10,000. The membranes were scanned with an ImageQuant LAS 4000 (GE Healthcare (Little Chalfont, Buckinghamshire, UK).) and analysed used ImageJ software (NIH) [16].

### 2.8. Flow Cytometry

The flow cytomery was performed according to standard protocol from previous studies [16,19] by using F4/80 (FITC), CD86 (PE) and CD206 (APC). Cells were separated into different tubes and incubated with Cytofix and Cytoperm. Next cells were stained with CD86 and CD206 according to the manufacturer’s directions. The samples were tested using a flow cytometer (Beckman CytoFLEX), and the results were analysed using CytExpert, and calculated by MFI.

The membrane expression of EP4 and GRK2 in RAW (M0, M1, M2): The flow cytomery was performed according to standard protocol from previous studies [16,19]. RAWs were separated into different tubes and stained with anti-mouse EP4 antibody (1:100) or anti-mouse GRK2 antibody (1:100) for 1 h. Followed by incubated with anti-mouse Alexa Fluor 488 for 30 min. The samples were tested using a flow cytometer (CytoFLEX, Beckman, Miami, FL, USA), and the results were analysed using CytExpert, and calculated by MFI.

### 2.9. Histological Examination

The ankle joints of mice were immersed in 10% formalin, then decalcified and paraffin-embedded [21]. The sections (5 mm) were stained with hematoxylin and eosin.

### 2.10. Confocal Microscopy

RAW culture was conducted in a 24-well plate with a glass plate attached at the bottom, fixed with 4% paraformaldehyde and blocked with 0.5% BSA [16]. The cells were incubated with GRK2 (1:100) and EP4 (1:100) antibody overnight in 4 °C. The secondary antibody were anti-rabbit Alexa Fluor 488 and anti-mouse Alexa Fluor 594 for 1 h at 37 °C. Fluorescent images were observed by a TCS SP8 confocal microscope (Leica, Wetzlar, Germany).

### 2.11. Small Interfering RNA (siRNA)

GRK2 siRNAs were chemically synthesized by Genepharma Co., Ltd. (Shanghai, China) to knockdown GRK2 in RAW macrophages as follows:

Negative control:

Sense: 5′-GUGAAUGCAGCUGACGCUUTT-3′

Anti-sense: 5′-AAGCGUCAGCUGCAUUCACTT-3′

GRK2-mus-738:

Sense: 5′-GGAAGAAUGUUGAGCUCAATT-3′

Anti-sense: 5′-UUGAGCUCAACAUUCUUCCTT-3′

RAW macrophages were transfected at 70% confluency in 6-well plates using Lipofectamine 2000 (Invitrogen, Carlsbad, CA, USA) according to the manufacturer’s protocol. After 48 h, deletion of GRK2 was confirmed via western blots (Supplementary Figure S4D).

### 2.12. RAW KO Cell Lines and Complementation

All Raw264.7 KO cell lines were created using the CRISPR/Cas9 system [20]. Target gRNA sequences were generated using CRISPR Design and are listed below. The deletion of GRK2 was confirmed via western blots (Supplementary Figure S4E). Non-targeting gRNA sequence: CGCTTCCGCGGCCCGTTCAA. The target gRNA sequences as follows (Table 1).

### 2.13. Co-Immunoprecipitation and QPCR

The Co-immunoprecipitation (CO-IP) was carried out according to standard protocol from previous studies [16]. Quantitative PCR was carried out with SYBR Green, as described previously [22]. The sequence of the primers for the EP receptors as follows:

EP1 (Forward): GTGAAGTGCGGGTGGAGG

EP1 (Reverse): AAGAGGGCCAGTGCTGCT

EP2 (Forward): GCTCCTTGCCTTTCACAATCTT

EP2 (Reverse): CAGGACCGGTGGCCTAAGTA

EP3 (Forward): TTCTGCCAGATCAGAGACCAC

EP3 (Reverse): ATCTTTCCAGCTGGTCACTCC

EP4 (Forward): GCTTGACAAGTTCCGCACTG

EP4 (Reverse): ATGGTACCTGTAGGGTGGGG

### 2.14. Statistical Analysis

Results are shown as means ± SEM. One-way analysis of variance was used to analyse the data from multiple groups. A t test was applied for comparisons between two groups. A *P* value < 0.05 was regarded as a significant difference. The data were obtained from three repeated experiments.

## 3. Results

### 3.1. The Imbalance of Macrophage Polarization in PMs, BMMs and SMs of CIA Mice

**Figure 1:**
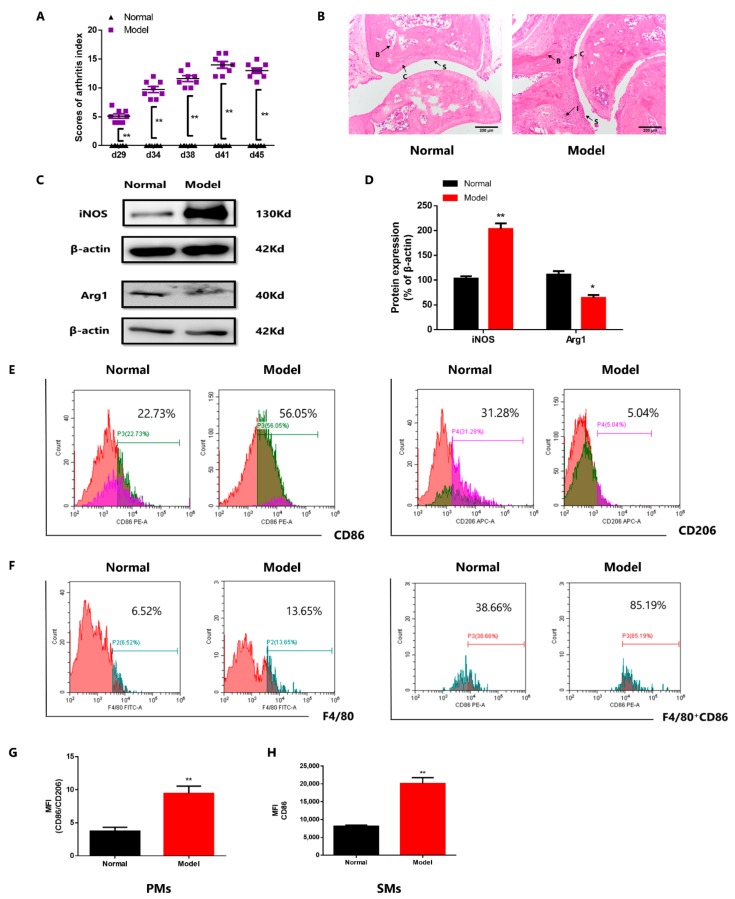
The imbalance of macrophage polarization in PMs, BMMs and SMs of CIA mice. (**A**) The polyarthritis index of CIA mice. Values are means ± SEM, with 8 mice/group. ** *P* < 0.01 vs. normal. (**B**) Representative micrographs of haematoxylin and eosin (H&E)-stained histological sections of the joints are shown. Original magnification × 200. The histology section shows the synoviocytes (S), the bone (**B**), and the cartilage (**C**). 4 mice/group. (**C**) The expression of iNOS and Arg1 in BMMs of CIA mice. (**D**) The expression of iNOS and Arg1 in BMMs of CIA mice. Values are means ± SEM, with 3 mice/group. * *P* < 0.05, ** *P* < 0.01 vs. normal. (**E**) The ratio of CD86/CD206 in PMs of CIA mice. Red population: Targeted cell population. Green population: CD86^+^ cells. Purple population: CD206^+^ cells. (**F**) The expression of CD86 in F4/80^+^ SMs of CIA mice. Red population in F4/80 figure: Targeted cell population. Green population: F4/80^+^ cells. Red population in F4/80^+^CD86 figure: CD86^+^ cells. (**G**) The ratio of CD86/CD206 in PMs of CIA mice. Values are means ± SEM, with 5 mice/group. ** *P* < 0.01 vs. normal. (**H**) The expression of CD86 in F4/80^+^ SMs of CIA mice. Values are means ± SEM, with 9–12 mice/group. *P* < 0.01 vs. normal.

We established CIA mice model and the polyarthritis index significantly increased after modeling, and reached peak at d41 (Figure 1A). We investigated the pathological severity of CIA ankle joints using H&E staining (Figure 1B). The synovial membrane structure was intact in the normal group. However, hyperplasia of synovial cell layer, inflammatory cell infiltration and cartilage injury were observed in CIA group.

A series of previous reports proven that the imbalance of macrophages polarization in RA [1,2,3,4,23,24]. Therefore, we evaluated the expression of M1 macrophages marker, iNOS and CD86, M2 macrophages marker, arginase-1 (Arg1) and CD206 in BMMs, PMs and SMs of CIA mice. The proportions of cultured PMs and BMMs are more than 90% (Supplementary Figure S1). Compared to the normal group, in the CIA group, the expression of iNOS upregulated and Arg1 downregulated in cultured BMMs (Figure 1C,D), the ratio of CD86/CD206 in cultured PMs increased (Figure 1E,G), and the expression of CD86 in F4/80^+^SMs augmented (Figure 1F,H). These results suggested that pro-inflammatory macrophages predominate in CIA mice.

### 3.2. Abnormal PGE2-cAMP-CREB Transduction in CIA Mice

**Figure 2:**
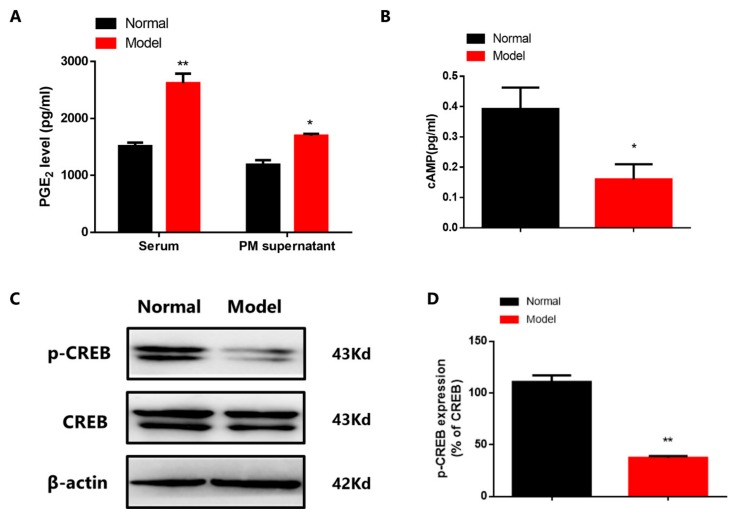
Abnormal PGE2-cAMP-CREB signaling in CIA mice. (**A**) The level of PGE2 in serum and PMs supernatant. Values are mean ± SEM, with six mice/group. * *P* < 0.05, ** *P* < 0.01 vs. normal. (**B**) The level of cAMP in PMs of CIA mice. Values are mean ± SEM, with six mice/group. * *P* < 0.05 vs. normal. (**C**) The level of p-CREB in PMs of CIA mice. (**D**) The expression of p-CREB in PMs of CIA mice. Values are mean ± SEM, with three mice/group. ** *P* < 0.01. vs. normal.

PMs can reflect the overall situation of the physiology and pathology situation, therefore, we mainly used PMs for further mechanism study. PGE2 can augment M2 polarization via activated cAMP-CREB pathway by paracrine and autocrine [7]. However, we observed that in CIA group, the level of PGE2 in serum and PMs supernatant increased (Figure 2A). Then we found the decreased expression of cAMP (Figure 2B,C) and p-CREB of PMs in CIA mice (Figure 2D), suggesting that the abnormal PGE2-cAMP-CREB signaling may induce the imbalance macrophage polarization of CIA mice.

### 3.3. Abnormal PGE2-cAMP-CREB Signaling Induced Macrophage Imbalance in CIA Mice

**Figure 3:**
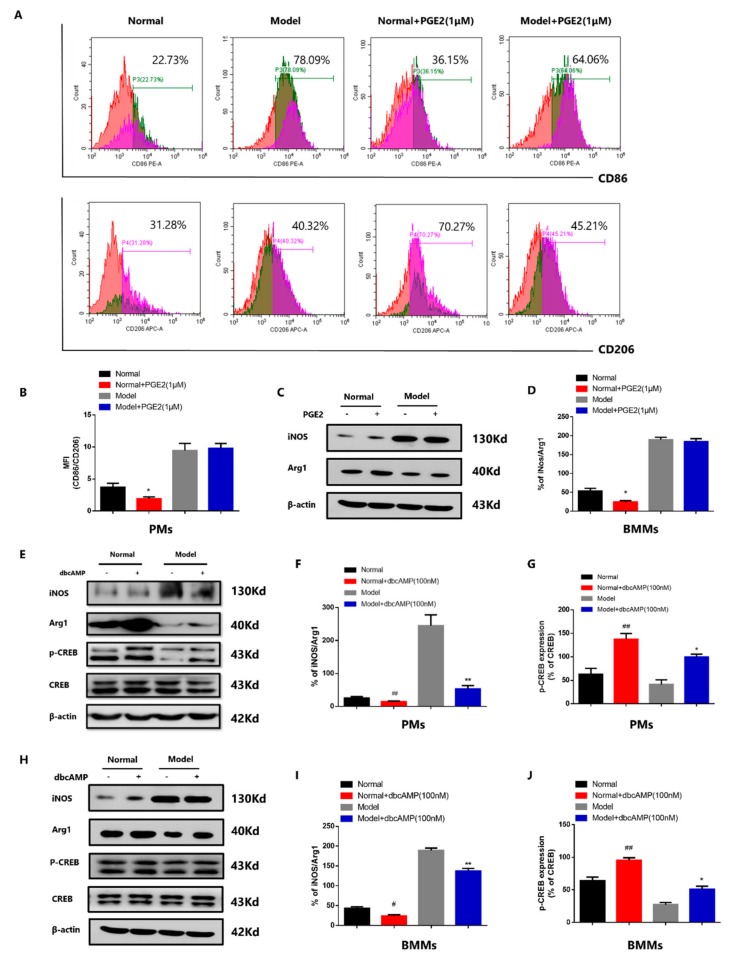
Abnormal PGE2-cAMP-CREB signaling induced macrophage imbalance in CIA mice. (**A**) The role of PGE2 on the ratio of CD86/CD206 in PMs. Red: Targeted cell population. Green: CD86+ cells. Purple: CD206+ cells. (**B**) The role of PGE2 on the ratio of CD86/CD206 in PMs. Values are means ± SEM, with three mice/group. * *P* < 0.05 vs. normal. (**C**) The role of PGE2 on the ratio of iNOS/Arg1 in BMMs. “+” means that the cells were grown in the presence of PGE2, “−” means that the cells were grown without PGE2. (**D**) The role of PGE2 on the ratio of iNOS/Arg1 in BMMs. Values are means ± SEM, with three mice/group. * *P* < 0.05 vs. normal. (**E**) The role of dbcAMP on the ratio of iNOS/Arg1 and the expression of p-CREB in PMs of CIA mice. “+” means that the cells were grown in the presence of dbcAMP, “−” means that the cells were grown without dbcAMP. (**F**) The role of dbcAMP on the ratio of iNOS/Arg1 in PMs. Values are means ± SEM, with three mice/group. ## *P* < 0.01 vs. normal, ** *P* < 0.01 vs. model. (**G**) The role of dbcAMP on the expression of p-CREB in PMs. Values are means ± SEM, with three mice/group. ## *P* < 0.01 vs. normal, * *P* < 0.05 vs. model. (**H**) The role of dbcAMP on the ratio of iNOS/Arg1 and the expression of p-CREB in BMMs of CIA mice. “+” means that the cells were grown in the presence of dbcAMP, “−” means that the cells were grown without dbcAMP. (**I**) The role of dbcAMP on the ratio of iNOS/Arg1 in BMMs. Values are means ± SEM, with three mice/group. # *P* < 0.05 vs. normal, ** *P* < 0.01 vs. model. (**J**) The role of dbcAMP on the expression of p-CREB in BMMs. Values are means ± SEM, with three mice/group. ## *P* < 0.01 vs. normal, * *P* < 0.05 vs. model.

To further evaluate the role of PGE2-cAMP-CREB signaling in macrophage polarization of CIA mice, we used PGE2 (1 μM, 1 h) and the cAMP agonist dbcAMP (100 nM, 1 h) to stimulate PMs both in normal group and CIA group. PGE2 (1 μM) decreased the CD86/CD206 ratio of PMs in normal group but did not change the CD86/CD206 ratio in model group (Figure 3A,B). We also confirmed the results in MCSF-induced BMMs. PGE2 (1 μM) decreased the iNOS/Arg1 ratio of BMMs in normal group but did not change the iNOS/Arg1 ratio in model group (Figure 3C,D). In CIA group, PGE2 did not promote M2 polarization may be related to its increased level and the increase of M1 macrophages. Next we respectively induced RAW into M1 and M2 macrophages, and assumed RAW without induction as M0 macrophage. Under the stimulation of different concentration (10 nM, 100 nM, 1 μM, 10 μM) of PGE2 (t = 24 h), the variation trend of the three subtypes of macrophages was different.

In PGE2-stimulated M0 macrophages, PGE2 (100 nM, 1 μM) decreased the ratio of CD86/CD206, while PGE2 (10 nM, 10 μM) did not change the ratio (Supplementary Figure S2A). In PGE2-stimulated M1 macrophages, PGE2 (1 μM, 10 μM) increased the ratio of CD86/CD206, while PGE2 (10 nM, 100 nM) did not change the ratio (Supplementary Figure S2B). In PGE2-stimulated M2 macrophages, PGE2 (10 nM, 100 nM, 1 μM, 10 μM) decreased the ratio of CD86/CD206 (Supplementary Figure S2C). Interestingly, the dbcAMP downregulated the ratio of iNOS/Arg1 both in PMs (Figure 3E,F) and BMMs (Figure 3H,I) in normal and CIA group, as well as the increased expression of p-CREB in PMs (Figure 3E,G) and BMMs (Figure 3H,J). The dbcAMP also exerted similar effect in M0, M1 and M2 macrophages, decreasing the ratio of CD86/CD206 (Supplementary Figure S2D).

### 3.4. The EP4 Over-Desensitization Resulted in the Abnormal Transduction of PGE2-cAMP-CREB Signaling in CIA Mice

**Figure 4:**
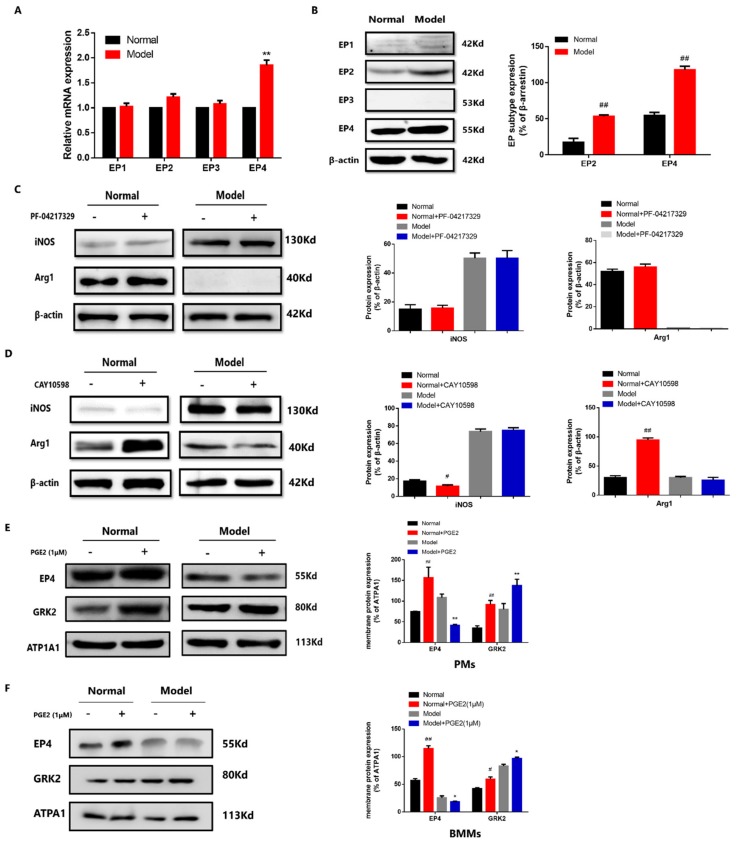
The EP4 over-desensitization resulted in the abnormal transduction of PGE2-cAMP-CREB signaling in CIA mice. (**A**) EP subtype mRNA expression in PMs of CIA mice. Values are mean ± SEM, with three mice/group. ** *P* < 0.01 vs. normal. (**B**) EP subtype protein expression in PMs of CIA mice. Values are means ± SEM, with three mice/group. ## *P* < 0.01 vs. normal. (**C**) The expression of iNOS and Arg1 in PF-04217329-stimulated PMs of CIA mice. Values are mean ± SEM, with three mice/group. “+” means that the cells were grown in the presence of PF-04217329, “−” means that the cells were grown without PF-04217329. (**D**) The expression of iNOS and Arg1 in CAY10598-stimulated PMs of CIA mice. Values are mean ± SEM, with three mice/group. # *P* < 0.05, ## *P* < 0.01 vs. normal. * *P* < 0.05 vs. model. “+” means that the cells were grown in the presence of CAY10598, “−” means that the cells were grown without CAY10598. (**E**) Membrane localization of EP4 and GRK2 in PGE2 stimulated-PMs of CIA. Values are mean ± SEM, with three mice/group. ## *P* < 0.01 vs. normal. ** *P* < 0.01 vs. Model. “+” means that the cells were grown in the presence of PGE2, “−” means that the cells were grown without PGE2. (**F**) Membrane localization of EP4 and GRK2 in PGE2 stimulated-BMMs of CIA. Values are mean ± SEM, with three mice/group. # *P* < 0.05, ## *P* < 0.01 vs. normal. * *P* < 0.05 vs. Model. “+” means that the cells were grown in the presence of PGE2, “−” means that the cells were grown without PGE2.

PGE2 and dbcAMP played different roles on PMs polarization in normal and CIA group, which may be related to the status of EP receptors sensitivity of macrophages. We detected the expression of EP1-EP4 receptors of PMs by western blot and QRT-PCR. The protein expression of EP2 increased (Figure 4A), and the protein expression (Figure 4A) and mRNA expression (Figure 4B) of EP4 were both significantly increased in the CIA group. To further investigate the role of EP2 and EP4 on macrophage polarization in CIA mice, we used EP2 agonist PF-04217329 (1 μM, 1 h) and EP4 agonist CAY10598 (1 μM, 1 h) to respectively stimulate PMs in normal group and CIA group. EP2 agonist PF-04217329 had no significant role on the expression of iNOS and Arg1 both in normal group and CIA group (Figure 4C). EP4 agonist CAY10598 downregulated the expression of iNOS and upregulated the expression of Arg1 in the PMs of normal group, but did not change the expression of iNOS and Arg1 in CIA group (Figure 4D). EP4 agonist had similar effects with PGE2 on the ratio of M1/M2 markers. Above results suggested that PGE2 regulated PMs polarization through EP4. Then we assessed whether the abnormal transduction of PGE2-EP4 signaling in CIA mice is related to the over-desensitization of EP4. Here we came back to test the membrane localization of EP4 in PGE2-stimulated PMs and BMMs. The membrane localization of EP4 increased in PMs (Figure 4E) and BMMs (Figure 4F) of normal group but decreased in CIA group. We also tested the EP4 membrane localization in PGE2-stimulated RAW. In PGE2-stimulated M0 macrophages, PGE2 (100 nM, 1 μM) increased the EP4 membrane colocalization, while PGE2 (10 nM, 10 μM) did not change the EP4 membrane colocalization (Supplementary Figure S3A). In PGE2-stimulated M1 macrophages, PGE2 (1 μM, 10 μM) decreased the EP4 membrane colocalization, while PGE2 (10 nM, 100 nM) did not change the EP4 membrane colocalization (Supplementary Figure S3B). In PGE2-stimulated M2 macrophages, PGE2 (10 nM, 100 nM, 1 μM, 10 μM) increased the EP4 membrane colocalization (Supplementary Figure S3C). The change of EP4 membrane localization in PGE2-stimulated PMs, BMMs and RAW was related to the macrophage polarization. GRK2 can phosphorylate agonist-activated EP4, triggering the binding of β-arrestin to the receptor, which induces EP4 desensitization [15,25]. We have demonstrated that the increase of GRK2 transferring to membrane induced the over-desensitization of EP4, as well as the abnormal transduction of PGE2-EP4-cAMP in FLS of AA rats [15]. Therefore, in order to verify the role of GRK2 on EP4 in PMs and RAW, we also detected the membrane localization of GRK2 in PGE2-stimulated PMs, BMMs and RAW. The membrane localization of GRK2 increased both in normal group and CIA group of PMs (Figure 4E) and BMMs (Figure 4F). Similarly, in the PGE2-stimulated RAW group, the membrane localization of GRK2 also gradually increased in PGE2 (1 μM, 10 μM)-stimulated M0 macrophages (Supplementary Figure S3D), PGE2 (10 nM, 100 nM, 1 μM, 10 μM)-stimulated M1 macrophages (Supplementary Figure S3E), and PGE2 (100 nM, 1 μM, 10 μM)-stimulated M2 macrophages (Supplementary Figure S3F).

### 3.5. GRK2-Induced Over-Desensitization of EP4 Involved in Macrophage Polarization

**Figure 5:**
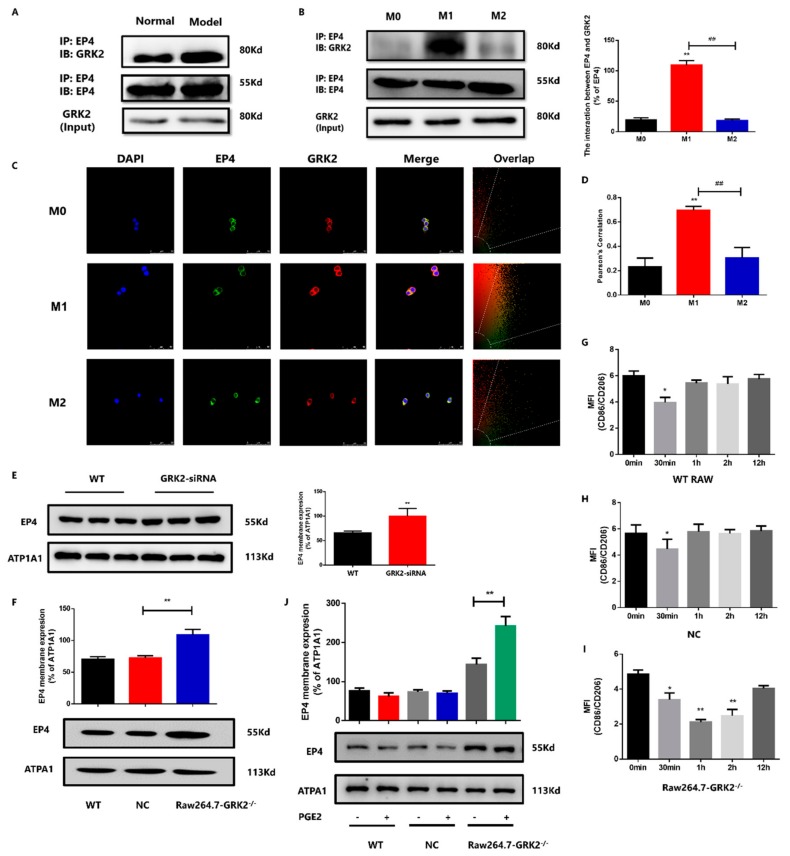
GRK2-induced over-desensitization of EP4 involved in macrophage polarization. (**A**) Co-expression of GRK2 and EP4 in PMs. With three mice/group. (**B**) Co-expression of GRK2 and EP4 in RAW (M0, M1 and M2 macrophages). Values are mean ± SEM, with three datapoints/group. ** *P* < 0.01 vs. M0. ## *P* < 0.01 vs. M1. (**C**) The co-localization of GRK2 and EP4 in RAW (M0, M1 and M2 macrophages). Original magnification × 50. (**D**) The Pearson’s correlation of GRK2 and EP4 in RAW (M0, M1 and M2 macrophages). Values are mean ± SEM, with three samples/group. ** *P* < 0.01 vs. M0. ## *P* < 0.01 vs. M1. (**E**) The membrane localization of EP4 in GRK2 siRNA-transfected RAW. Values are mean ± SEM, with three datapoints/group. ** *P* < 0.01 vs. WT. (**F**) The membrane localization of EP4 in Raw264.7-GRK2^−/−^. Values are mean ± SEM, with three datapoints/group. ** *P* < 0.01 vs. NC. (**G**) The ratio of CD86/CD206 in WT RAW treated by PGE2 at different time. Values are mean ± SEM, with three datapoints/group. * *P* < 0.05 vs. 0 min. (**H**) The ratio of CD86/CD206 in NC RAW treated by PGE2 at different time. Values are mean ± SEM, with three datapoints/group. * *P* < 0.05 vs. 0 min. (**I**) The ratio of CD86/CD206 in Raw264.7-GRK2^−/−^ treated by PGE2 at different time. Values are mean ± SEM, with three datapoints/group. * *P* < 0.05, ** *P* < 0.01 vs. 0 min. (**J**) The membrane localization of EP4 in PGE2-stimulated WT RAW, NC RAW and Raw264.7-GRK2^−/−^. Values are mean ± SEM, with three datapoints/group. ** *P* < 0.01 vs. untreated group.

To further detect the role of GRK2 on EP4 over-desensitization, we used CO-IP to detect the interaction between GRK2 and EP4. The interaction between EP4 and GRK2 in PMs increased, which may relate to increased GRK2 translocation to membrane and phosphorylated EP4 in CIA mice (Figure 5A). Compared to M0 macrophages, the interaction between EP4 and GRK2 increased in M1 macrophages. There was no significant change between M0 and M2 macrophages. In addition, compared to M1 macrophages, the interaction between EP4 and GRK2 decreased in M2 macrophages (Figure 5B). In laser confocal microscopy experiment, the pearson’s correlation (indicating the binding capacity between proteins) increased in M1 and showed no significant change in M2 macrophages (Figure 5C,D). The results of CO-IP experiment are consistent with those of laser confocal microscopy experiment. Next, our results with the use of GRK2 siRNAs or CRISPR/Cas9 demonstrated that compared to control group, the membrane localization of EP4 increased in GRK2-siRNA group (Figure 5E) and Raw-GRK2^−/−^ group (Figure 5F). We stimulated WT RAW and negative control (NC) group with PGE2 (10 μM) at different times to simulate over-desensitization. The NCs are non-targeting gRNA sequences. The ratio of CD86/CD206 decreased at 30 min and did not change at other time both in WT RAW (Figure 5G and Supplementary Figure S4A) and NC RAW (Figure 5H and Supplementary Figure S4B). PGE2 (10 μM) were also used to stimulate Raw-GRK2^−/−^ cells at different times. The ratio of CD86/CD206 decreased at 30 min, 1 h and 2 h (Figure 5I and Supplementary Figure S4C). Then we chose PGE2 to stimulate WT RAW, NC RAW and Raw-GRK2^−/−^ cells for 1 h, the membrane localization of EP4 in PGE2-stimulated WT RAW and NC RAW did not change significantly, but increased in PGE2-stimulated Raw-GRK2^−/−^ cells (Figure 5J). Above results indicated that the deletion of GRK2 resulted a higher EP4 sensitivity when PGE2 stimulated RAW.

### 3.6. The Deletion of GRK2 Promoted M2 Polarization through cAMP-CREB Pathway

**Figure 6:**
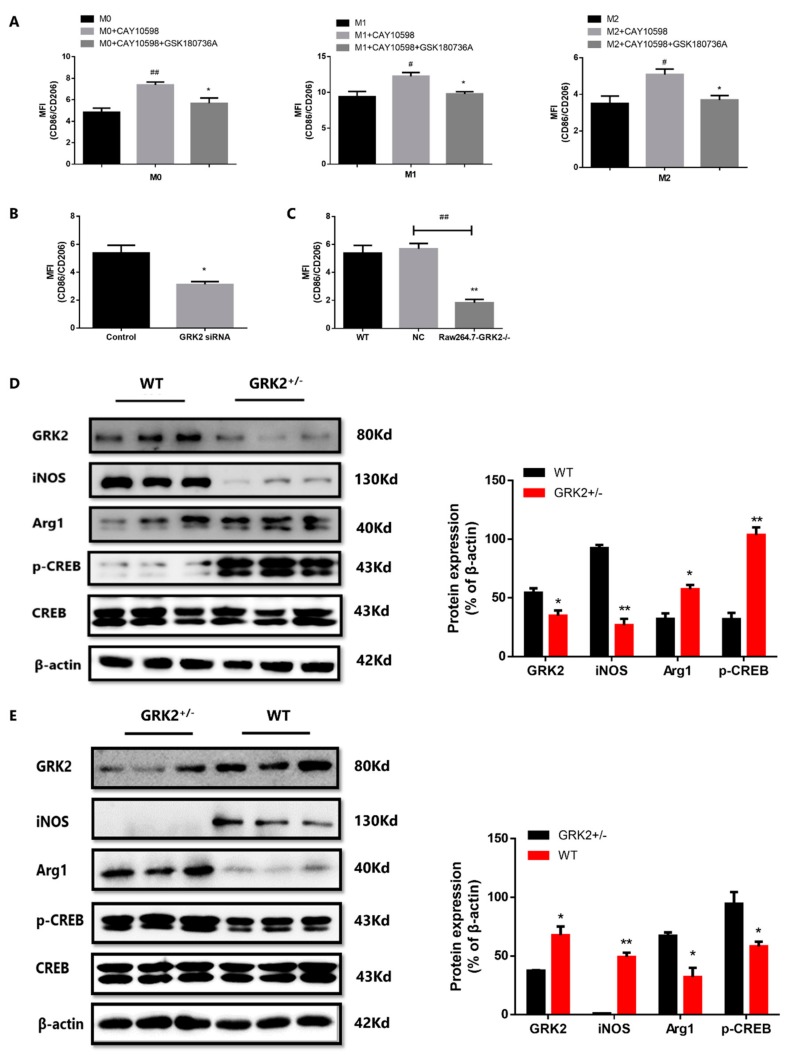
The deletion of GRK2 promoted M2 polarization through cAMP-CREB pathway. (**A**) The role of GRK2 inhibitor on Raw (M0, M1 and M2 macrophages). Values are means ± SEM, with four datapoints/group. # *P* < 0.05, ## *P* < 0.01 vs. M0, M1 or M2. * *P* < 0.05 vs. CAY10598-treated group. (**B**) The role of GRK2 siRNA on macrophage polarization. Values are means ± SEM, with four datapoints/group. * *P* < 0.05 vs. control. (**C**) The ratio of CD86/CD206 in Raw264.7-GRK2^−/−^. Values are means ± SEM, with four datapoints/group. ## *P* < 0.01 vs, NC. (**D**) The expression of iNOS, Arg1 and p-CREB in BMMs of GRK2^+/−^ mice. Values are mean ± SEM, with three mice/group. * *P* < 0.05, ** *P* < 0.01 vs. WT. (**E**) The expression of iNOS, Arg1 and p-CREB in PMs of GRK2^+/−^ mice. Values are mean ± SEM, with three mice/group. * *P* < 0.05, ** *P* < 0.01 vs. WT.

To determine the role of GRK2 on EP4 and macrophages polarization, we used EP4 agonist CAY10598 (1 μM) and GRK2 inhibitor GSK180736A (2 μM) to stimulate RAW at the same time for 24 h. The ratio of CD86/CD206 significantly increased when exposure to EP4 agonist, while decreased in GRK2 inhibitor-treated group (Figure 6A and Supplementary Figure S5A). Then we silenced GRK2 in RAW through siRNA, compared to un-transfected group, the ratio of CD86/CD206 decreased in GRK2-siRNA transfected RAW (Figure 6B and Supplementary Figure S5B). We further used CRISPR/Cas9 to knock out the GRK2 in RAW, compared to NC RAW, the ratio of CD86/CD206 significantly decreased in Raw-GRK2^−/−^ cells (Figure 6C and Supplementary Figure S5C). We also designed GRK2^+/−^ mice to further study the effect of GRK2 on macrophages polarization. GRK2 gene located in mouse 19# chromosome. Reportedly, the homozygous mice with GRK2 gene knockout died in embryo at 10.5 and 11.5 days. Therefore, the GRK2^+/−^ mice knocked down by CRISPR/Cas9 are heterozygous. We cultured primary BMMs and PMs of GRK2^+/−^ mice and detected the expression of GRK2. Compared to WT mice, the expression of GRK2 in BMMs (Figure 6D) and PMs (Figure 6E) significantly decreased in GRK2^+/−^ mice. We also found the GRK2 expression in spleen, thymus and heart of GRK2^+/−^ mice was lower that of WT mice (unpublished). Compared to WT mice, the expression of iNOS significantly decreased, the expression of Arg1 and p-CREB significantly increased in the cultured BMMs and PMs from GRK2^+/−^ mice (Figure 6D,E). In summary, we interpreted these observations to indicate that GRK2-induced over-desensitization of EP4 suppressed the normal transduction of PGE2-cAMP-CREB signaling; following by abnormal PGE2-cAMP-CREB signaling promoted M1 macrophage polarization in CIA mice.

## 4. Discussion

Extensive research into RA has identified the importance of macrophages in the pathogenesis of RA [24,26]. It has been previously reported that the PGE2/EPs system is essential for macrophage polarization. Minami M et al. demonstrated that PGE2 markedly suppressed production of several chemokines in LPS-stimulated human primary macrophages through EP4 [27]. Bing et al. further indicated that PGE2 promoted M2 polarization part via cAMP-CREB pathway [7]. However, several studies observed the increased level of PGE2 but decreased M2 macrophages in RA patients and arthritis animal model, the exact reason has not been explained. Whether PGE2 acts as pro-inflammatory or anti-inflammatory remains controversial. The biological function of individual EP receptors in RA is complex and cannot be predicted from isolated in vitro experiments. The role of PGE2 on macrophage polarization should depend on the regulated cell type and cell state (physiological or pathological), EP receptor on cells and EP-mediated downstream signals.

In this work, we found the abnormal PGE2-EP4-cAMP-CREB signaling induced the increase of M1 macrophages in CIA group for the first time. First, we found that PGE2 (1 μM) suppressed the M1 macrophages of normal group while failed to work in CIA group. The dbcAMP, a cAMP analogue, promoted M2 macrophages polarization both in normal group and CIA group. In vitro experiment, the dbcAMP promoted M2 polarization in M0, M1 and M2 macrophages, while PGE2 (10 μM) failed to promote M2 macrophages polarization in M0 and M1 macrophages. These results suggested that the intermediate link between PGE2 and cAMP, EP receptor, is involved in different role of PGE2. Then we detected the protein expression and mRNA expression of EP subtype receptors and revealed that PGE2 might mediate the cAMP-CREB pathway through EP2 or EP4. We further used EP2 agonists and EP4 agonists and found that EP4 agonists had similar effects to PGE2. Consistent with our observations, macrophages express EP4 more abundantly than other PGE receptors [8,27].

We considered the mechanism to explain increased total expression of EP4 when induced decreased level of cAMP. In accordance with our previous finding, constant stimulant of PGE2 on FLS of AA rats resulted in decreased level of cAMP, which is primarily caused by GRK2-induced EP4 over-desensitization [15]. Although the total expression of EP4 increased in AA FLS, the EP4 over-desensitization resulted in the loss of sensitivity to PGE2. Therefore, we tested the membrane localization of EP4 in PGE2-stimulated macrophages. The membrane localization of EP4 increased in normal group while decreased in CIA group, which was in agreement with our previous reports in FLS, suggesting that the over-desensitization of EP4 was involved in macrophages polarization imbalance in CIA mice. Noticeably, nonsteroidal anti-inflammatory drugs, PGE2 synthesis inhibitor and other prostanoids, continue to be used in the treatment of RA [28]. However, PGE2 inhibitor was not ideal for RA treatment when the physiological function of PGE2 was blocked. In addition, PGE2 plays different roles in regulating biological and pathological functions, blindly to block or activate receptor cannot fundamentally solve problem, activating or blocking should depend on the desensitization condition of receptor.

The different role of GRK2 in the regulation of intracellular signaling depends on specific stimuli, cell type or physiological context [29], but also the subcellular localization is a fine predictor of its function. GRK2 regulates GPCR activation when it is localized on plasma membrane [30]. Our group has long been committed to the role of GRK2 in RA and arthritis animal models, here we investigated the effect of GRK2 on macrophage polarization in RA for the first time. We observed that the membrane localization of GRK2 increased in inflammatory macrophages, as well as the interaction between EP4 and GRK2. Next, we knock out GRK2 in RAW through siRNA or CRISPR/Cas9, the membrane localization of EP4 significantly increased in GRK2 KO RAW. Exposed to PGE2 (10 μM), the EP4 membrane localization increased in GRK2 KO RAW while did not change in untreated group. These results indicated that the reduction of GRK2 contributed to restore the sensitization of EP4.

In order to elucidate the role of GRK2 on macrophages polarization, we used EP4 agonist to stimulate RAW. These data indicated that EP4 agonist increased the CD86/CD206 ratio in M0, M1 and M2 macrophages, while the GRK2 inhibitor decreased the CD86/CD206 ratio in all groups. Here we did not test the effect of other GRKs on EP4, which will be considered in subsequent experiments. We also observed the decreased ratio of CD86/CD206 in GRK2 siRNAs and GRK2 KO RAW, which were related to their increased membrane localization of EP4. Then we generated GRK2 gene knockout mice using the CRISPR/Cas9 system and showed that compared to the WT mice, the expression of iNOS significantly decreased, and the expression of Arg1 and p-CREB significantly increased in PMs and BMMs of GRK2^+/−^ mice.

Due to the limited number and inaccessibility of SMs, the origin of SMs has not been fully illuminated. Indeed, SMs have been shown to play an important role in the pathogenesis of RA. In the present study, we explored the mechanism of macrophages polarization mainly through PMs and RAW. However, the role of locally released PGE2 in synovium on macrophage polarization is still not enough to explain. Recently, the origin of synovial macrophages has been revealed by our group [19]. Two types of SM were found: embryonic SMs (ESMs) which were F4/80^+^CD11b^−^ and appeared at a mid-embryonic stage; and bone marrow-derived SMs (BMSMs), which were F4/80^−^CD11b^+^ and appeared at a late-embryonic stage. ESMs expressed anti-inflammatory cytokines such as IL-4 and IL-10, and BMSMs expressed pro-inflammatory cytokines such as IL-1β and TNF. We will further explore the mechanism of the difference between the two types of macrophages. In summary, these data suggested that GRK2-induced EP4 over-desensitization suppressed the normal PGE2-cAMP-CREB signaling transduction and promoted M1 polarization (Graphic abstract). Due to the therapeutic potential of changing macrophage phenotype in RA, deeply investigated the mechanisms of polarization of macrophages plays an essential role in treating RA. Our results suggested a novel role for GRK2 in regulating macrophage polarization and provided new idea for precision treatment of RA.

In normal physiology, PGE2 promoted M2 polarization through cAMP-CREB pathway. While in CIA mice, the constant stimulus of PGE2 on EP4 induced increased GRK2 transferring to membrane, then leading to the over-desensitization of EP4. The abnormal transduction of PGE2-EP4 and its downstream signaling reduced the level of cAMP and p-CREB, which lead to the imbalance of macrophage polarization in CIA mice.

## Figures and Tables

**Table 1:** The target gRNA sequences using CRISPR Design.

NO.	5′	STEM	3′
Adrbk1-sgRNA(05985-2)-a	CACCg	CTTGGTGGAGTTCTACGAAG	
Adrbk1-sgRNA(05985-2)-b	aaac	CTTCGTAGAACTCCACCAAG	c
Adrbk1-sgRNA(05986-1)-a	CACCg	AGATTTGTCAGAACCTCCGA	
Adrbk1-sgRNA(05986-1)-b	aaac	TCGGAGGTTCTGACAAATCT	c
Adrbk1-sgRNA(05987-1)-a	CACCg	AGTCAAAGATCTCTCGGCTG	
Adrbk1-sgRNA(05987-1)-b	aaac	CAGCCGAGAGATCTTTGACT	c

## Supplementary Materials

The following are available online at https://www.mdpi.com/2073-4409/8/12/1596/s1, Figure S1: The proportion of PMs and BMMs., Figure S2: The role of PGE2 and dbcAMP on the ratio of CD86/CD206 in M0, M1 and M2. Figure S3: EP4 and GRK2 membrane expression in PGE2-stimulated RAW (M0, M1 and M2). Figure S4: The ratio of CD86/CD206 in RAW treated by PGE2 at different time. Figure S5: The role of GRK2 inhibitor, GRK2 siRNA and GRK2 knockout on M0, M1 and M2 macrophages.

## References

[1] Iwamoto T., Okamoto H., Toyama Y., Momohara S. (2008). Molecular aspects of rheumatoid arthritis: Chemokines in the joints of patients. FEBS J..

[2] Burmester G.R., Stuhlmüller B., Keyszer G., Kinne R.W. (1997). Mononuclear phagocytes and rheumatoid synovitis. Mastermind or workhorse in arthritis?. Arthritis. Rheum..

[3] Sack U., Stiehl P., Geiler G. (1994). Distribution of macrophages in rheumatoid synovial membrane and its association with basic activity. Rheumatol. Int..

[4] Haringman J.J., Gerlag D.M., Zwinderman A.H., Smeets T.J., Kraan M.C., Baeten D., McInnes I.B., Bresnihan B., Tak P.P. (2005). Synovial tissue macrophages: A sensitive biomarker for response to treatment in patients with rheumatoid arthritis. Ann. Rheum. Dis..

[5] Udalova I.A., Mantovani A., Feldmann M. (2006). Macrophage heterogeneity in the context of rheumatoid arthritis. Nat. Rev. Rheumatol..

[6] Miossec P., Kolls J.K. (2012). Targeting IL-17 and TH17 cells in chronic inflammation. Nat. Rev. Drug Discov..

[7] Luan B., Yoon Y.S., Le Lay J., Kaestner K.H., Hedrick S., Montminy M. (2015). CREB pathway links PGE2 signaling with macrophage polarization. Proc. Natl. Acad. Sci. USA.

[8] Wu H., Wei W., Song L., Zhang L., Chen Y., Hu X. (2007). Paeoniflorin induced immune tolerance of mesenteric lymph node lymphocytes via enhancing beta 2-adrenergic receptor desensitization in rats with adjuvant arthritis. Int. Immunopharmacol..

[9] Chen J.Y., Wu H.X., Chen Y., Zhang L.L., Wang Q.T., Sun W.Y., Wei W. (2012). Paeoniflorin inhibits proliferation of fibroblast-like synoviocytes through suppressing G-protein-coupled receptor kinase 2. Planta Med..

[10] Métayé T., Gibelin H., Perdrisot R., Kraimps J.L. (2005). Pathophysiological roles of G-protein-coupled receptor kinases. Cell. Signal..

[11] Singhmar P., Huo X., Eijkelkamp N., Berciano S.R., Baameur F., Mei F.C., Zhu Y., Cheng X., Hawke D., Mayor F. (2016). Critical role for Epac1 in inflammatory pain controlled by GRK2-mediated phosphorylation of Epac1. Proc. Natl. Acad. Sci. USA.

[12] Degos V., Peineau S., Nijboer C., Kaindl A.M., Sigaut S., Favrais G., Plaisant F., Teissier N., Gouadon E., Lombet A. (2013). G protein-coupled receptor kinase 2 and group I metabotropic glutamate receptors mediate inflammation-induced sensitization to excitotoxic neurodegeneration. Ann. Neurol..

[13] Wang Q., Wang L., Wu L., Zhang M., Hu S., Wang R., Han Y., Wu Y., Zhang L., Wang X. (2017). Paroxetine alleviates T lymphocyte activation and infiltration to joints of collagen-induced arthritis. Sci. Rep..

[14] Pfleger J., Gresham K., Koch W.J. (2019). G protein-coupled receptor kinases as therapeutic targets in the heart. Nat. Rev. Cardiol..

[15] Jia X.Y., Chang Y., Wei F., Dai X., Wu Y.J., Sun X.J., Xu S., Wu H.X., Wang C., Yang X.Z. (2019). CP-25 reverses prostaglandin E4 receptor desensitization-induced fibroblast-like synoviocyte dysfunction via the G protein-coupled receptor kinase 2 in autoimmune arthritis. Acta Pharmacol. Sin..

[16] Yang X., Zhao Y., Jia X., Wang C., Wu Y., Zhang L., Chang Y., Wei W. (2019). CP 25 combined with MTX/ LEF ameliorates the progression of adjuvant induced arthritis by the inhibition on GRK2 translocation. Biomed. Pharmacother..

[17] Chen J., Wang Y., Wu H., Yan S., Chang Y., Wei W. (2018). A Modified Compound from Paeoniflorin, CP-25, Suppressed Immune Responses and Synovium Inflammation in Collagen-Induced Arthritis Mice. Front. Pharmacol..

[18] Weischenfeldt J., Porse B. (2008). Bone marrow-derived macrophages (BMM): Isolation and applications. CSH Protoc..

[19] Tu J., Hong W., Guo Y., Zhang P., Fang Y., Wang X., Chen X., Lu S., Wei W. (2019). Ontogeny of synovial macrophages and the roles of synovial macrophages from different origins in arthritis. Front. Immunol..

[20] Cong L., Ran F.A., Cox D., Lin S., Barretto R., Habib N., Hsu P.D., Wu X., Jiang W., Marraffini L.A. (2013). Multiplex genome engineering using CRI SPR/Cas systems. Science.

[21] Shu J.L., Zhang X.Z., Han L., Zhang F., Wu Y.J., Tang X.Y., Wang C., Tai Y., Wang Q.T., Chen J.Y. (2019). Paeoniflorin-6′-O-benzene sulfonate alleviates collagen induced arthritis in mice by downregulating BAFF-TRAF2- NF-κB signaling comparison with biological agents. Acta Pharmacol. Sin..

[22] Screaton R.A., Conkright M.D., Katoh Y., Best J.L., Canettieri G., Jeffries S., Guzman E., Niessen S., Yates J.R., Takemori H. (2004). The CREB coactivator TORC2 functions as a calcium- and cAMP-sensitive coincidence detector. Cell.

[23] Ambarus C.A., Noordenbos T., de Hair M.J., Tak P.P., Baeten D.L. (2012). Intimal lining layer macrophages but not synovial sublining macrophages display an IL-10 polarized-like phenotype in chronic synovitis. Arthritis Res. Ther..

[24] Soler Palacios B., Estrada-Capetillo L., Izquierdo E., Criado G., Nieto C., Municio C., González-Alvaro I., Sánchez-Mateos P., Pablos J.L., Corbí A.L. (2015). Macrophages from the synovium of active rheumatoid arthritis exhibit an activin A-dependent proinflammatory profile. J. Pathol..

[25] Premont R.T., Gainetdinov R.R. (2007). Physiological roles of G protein-coupled receptor kinases and arrestins. Annu. Rev. Physiol..

[26] Apel F., Zychlinsky A., Kenny E.F. (2018). The role of neutrophil extracellular traps in rheumatic diseases. Nat. Rev. Rheumatol..

[27] Minami M., Shimizu K., Okamoto Y., Folco E., Ilasaca M.L., Feinberg M.W., Aikawa M., Libby P. (2008). Prostaglandin E receptor type 4-associated protein interacts directly with NF-kappaB1 and attenuates macrophage activation. J. Biol. Chem..

[28] McCoy J.M., Wicks J.R., Audoly L.P. (2002). The role of prostaglandin E2 receptors in the pathogenesis of rheumatoid arthritis. J. Clin. Investig..

[29] Penela P., Murga C., Ribas C., Lafarga V., Mayor Jr F. (2012). The complex G protein-coupled receptor kinase 2 (GRK2) interactome unveils new physiopathological targets. (Translated from eng). Br. J. Pharmacol..

[30] Pierce K.L., Premont R.T., Lefkowitz R.J. (2002). Seven-transmembrane receptors. Nat. Rev. Mol. Cell Biol..

