# Supplementary material for: GRK2 Mediated Abnormal Transduction of PGE2-EP4-cAMP-CREB Signaling Induces the Imbalance of Macrophages Polarization in Collagen-Induced Arthritis Mice
Source: Cells. 2019 Dec 8;8(12):1596. doi: 10.3390/cells8121596 (PMC6953022; doi:10.3390/cells8121596)
Supplement: Supplementary file 1 [file cells-08-01596-s001.pdf]

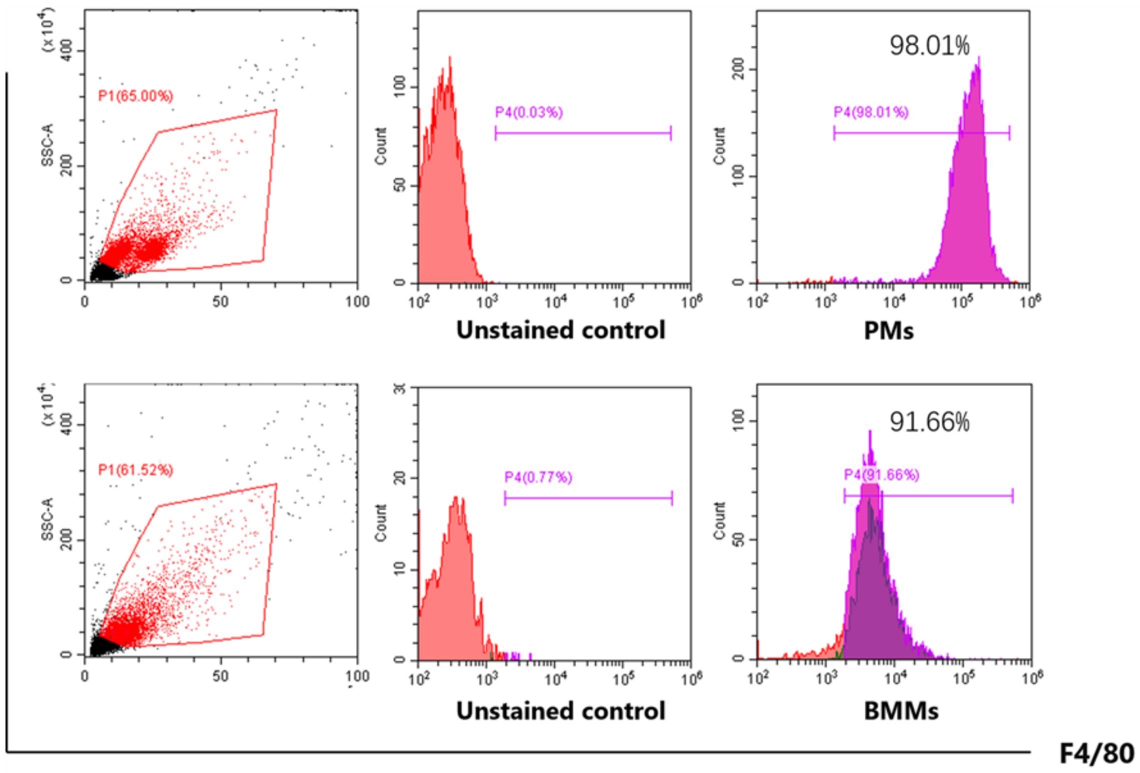

Supplementary Figure 1. The proportion of PMs and BMMs. Red population: Targeted cell population. Purple population: F4/80⁺ cells.

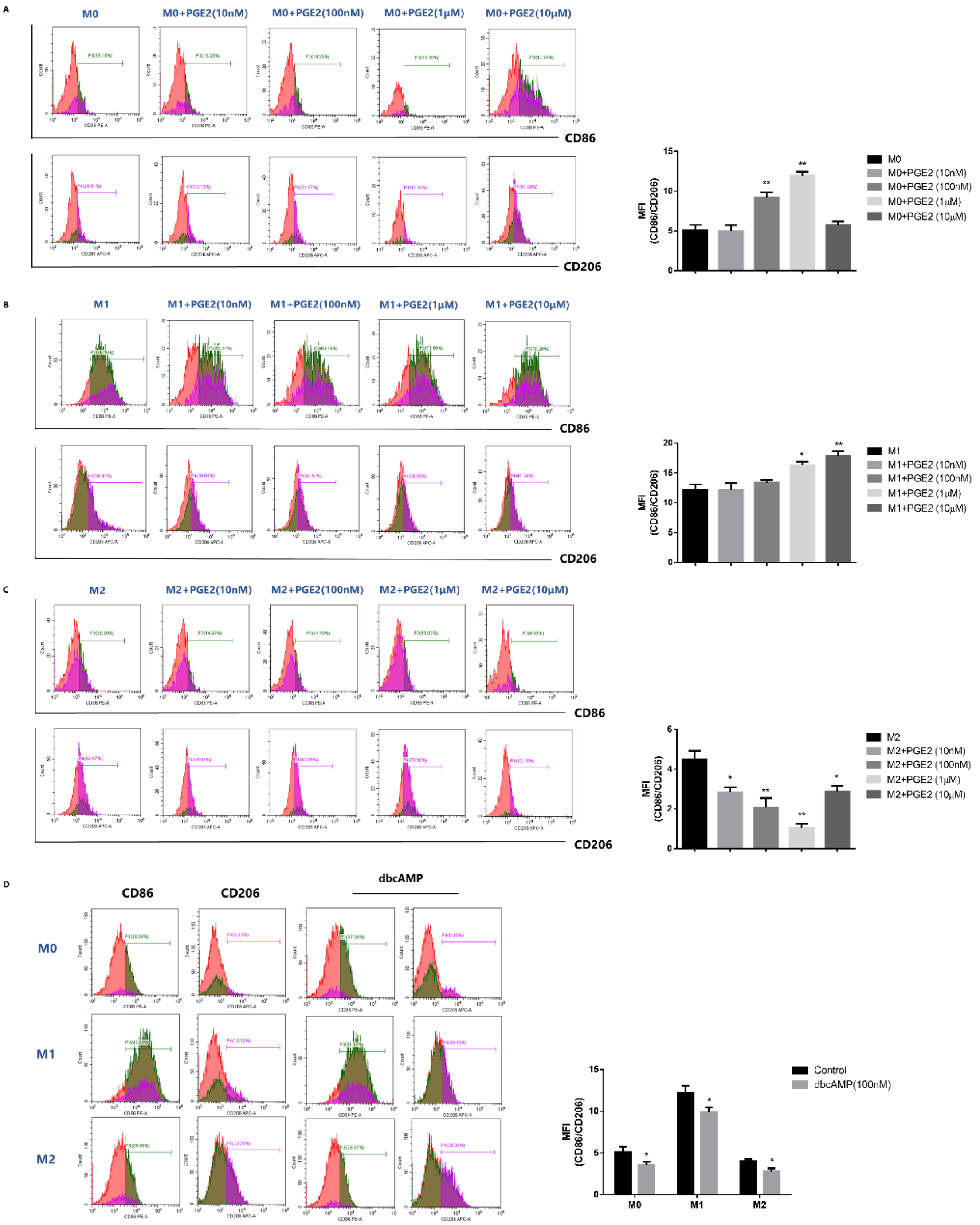

Supplementary Figure 2. In all figures, red population: Targeted cell population. Green population: $CD86^+$ cells. Purple population: $CD206^+$ cells. (A)The role of PGE2 on the ratio of CD86/CD206 in M0. Values are means ± SEM, with 4 data/group. * $P < 0.05$, ** $P < 0.01$ vs M0. (B) The role of PGE2 on the ratio of CD86/CD206 in M1. Values are means ± SEM, with 4 data/group. * $P < 0.05$, ** $P < 0.01$ vs M1. (C) The role of PGE2 on the ratio of CD86/CD206 in M2. Values are means ± SEM, with 4 data/group. * $P < 0.05$, ** $P < 0.01$ vs M2. (D) The role of dbcAMP on the ratio of CD86/CD206 in M0, M1 and M2. Values are means ± SEM, with 4 data/group. * $P < 0.05$ vs control.

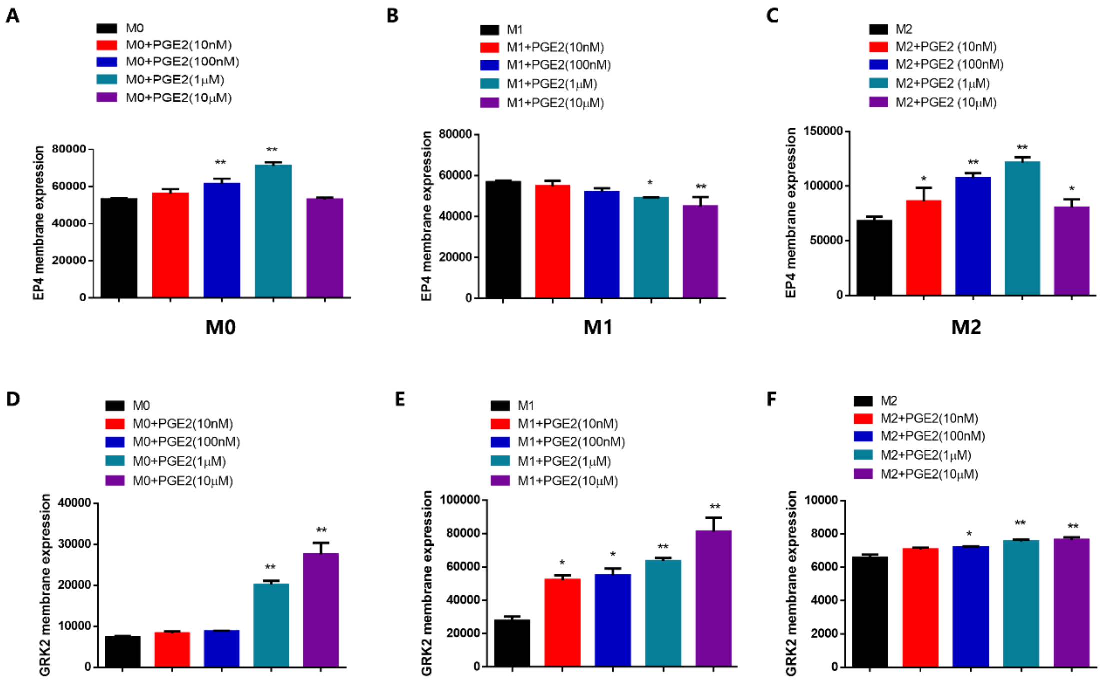

Supplementary Figure 3. EP4 and GRK2 membrane expression in PGE2-stimulated RAW (M0, M1 and M2). (A) EP4 membrane expression in PGE2-stimulated M0 macrophages. Values are means ± SEM, with 5 data /group. ** P < 0.01 vs M0. (B) EP4 membrane expression in PGE2-stimulated M1 macrophages. Values are means ± SEM, with 5 data /group. * P < 0.05, ** P < 0.01 vs M1. (C) EP4 membrane expression in PGE2-stimulated M2 macrophages. Values are means ± SEM, with 5 data /group. * P < 0.05, ** P < 0.01 vs M2. (D) GRK2 membrane expression in PGE2-stimulated M0 macrophages. Values are means ± SEM, with 5 data /group. * P < 0.05, ** P < 0.01 vs M0. (E) GRK2 membrane expression in PGE2-stimulated M1 macrophages. Values are means ± SEM, with 5 data /group. * P < 0.05, ** P < 0.01 vs M1. (F) GRK2 membrane expression in PGE2-stimulated M2 macrophages. Values are means ± SEM, with 5 data /group. ** P < 0.01 vs M2.

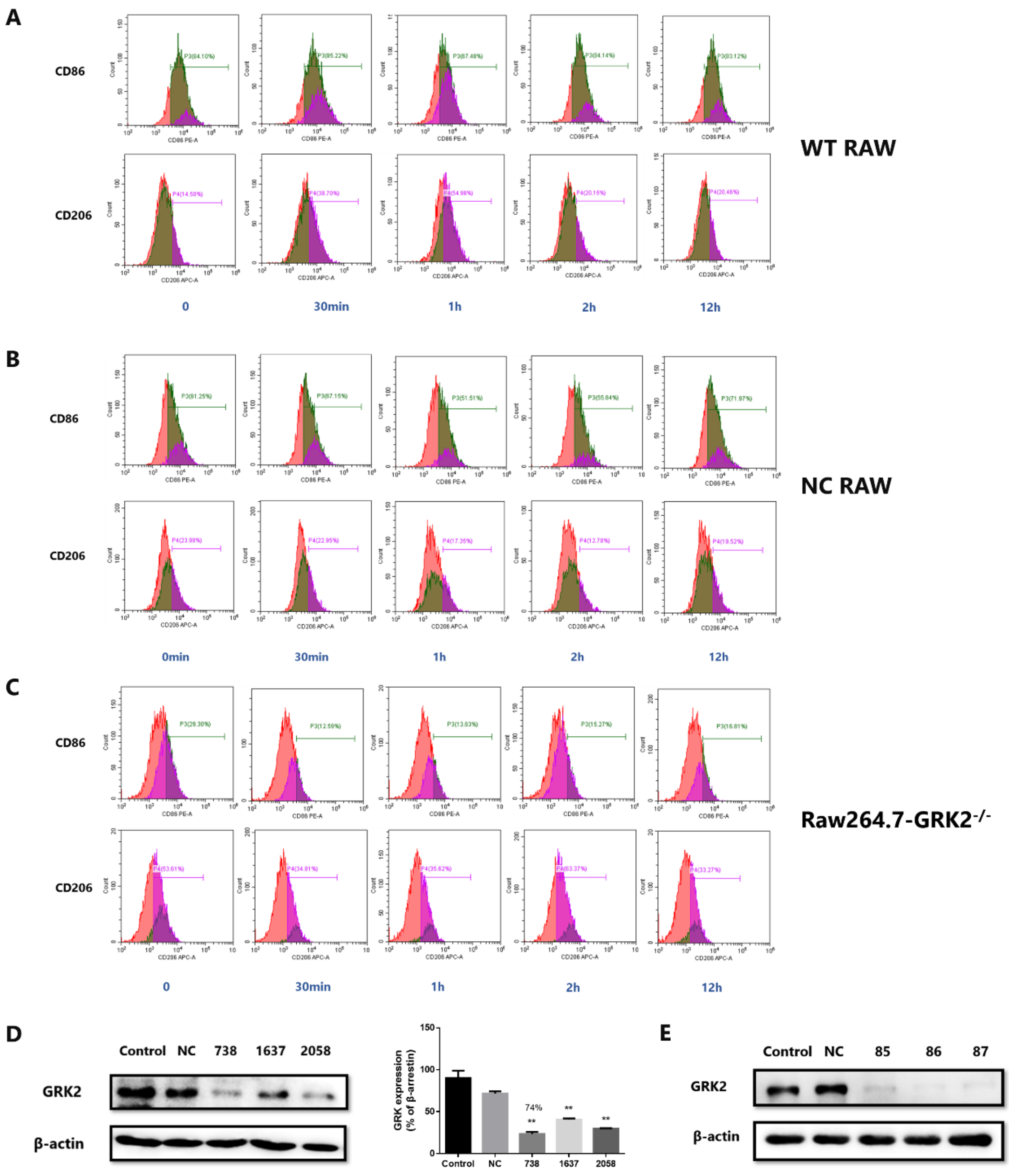

Supplementary Figure 4. In all figures, red population: Targeted cell population. Green population: $CD86^+$ cells. Purple population: $CD206^+$ cells. (A) The ratio of CD86/CD206 in WT RAW treated by PGE2 at different time. (B) The ratio of CD86/CD206 in NC RAW treated by PGE2 at different time. (C) The ratio of CD86/CD206 in Raw264.7-$GRK2^{-/-}$ treated by PGE2 at different time. (D) The GRK2 knockdown rate in GRK2 siRNA-transfected RAW. Values are mean ± SEM, with 3 data /group. ** $P < 0.01$ vs NC. (E) The GRK2 knockout rate in Raw264.7-$GRK2^{-/-}$. Values are mean ± SEM, with 3 data /group.

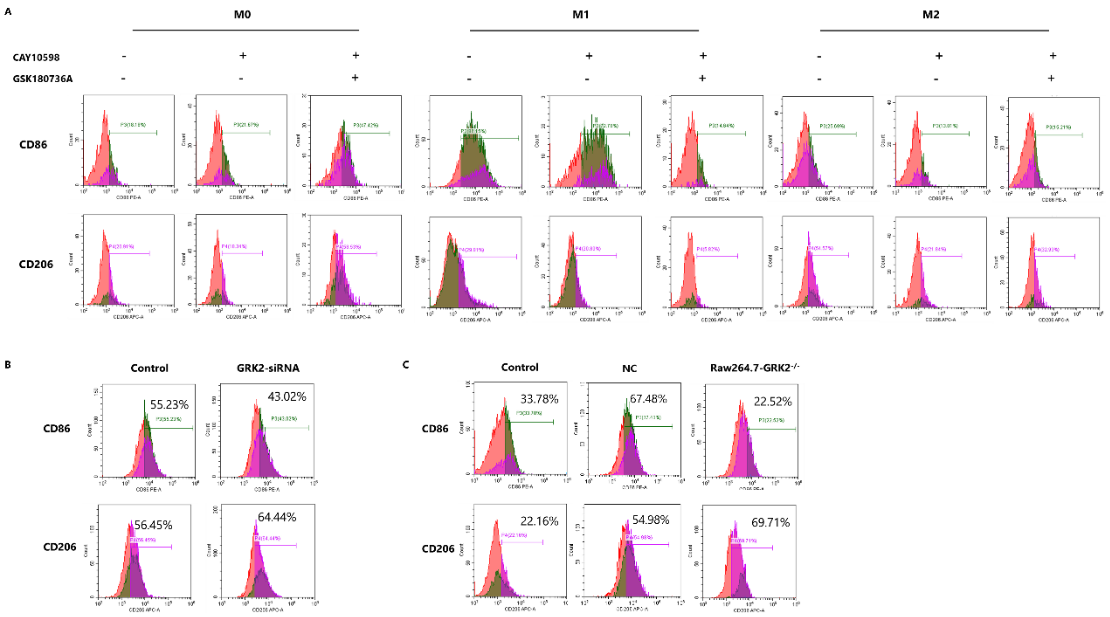

Supplementary Figure 5. In all figures, red population: Targeted cell population. Green population: $CD86^+$ cells. Purple population: $CD206^+$ cells. (A) The role of GRK2 inhibitor on M0, M1 and M2 macrophages. (B) The role of GRK2 siRNA on macrophage polarization. (C) The ratio of CD86/CD206 in Raw264.7-$GRK2^{-/-}$.
